# Effects of interstitial carbon atoms on texture structure and mechanical properties of FeMnCoCr alloys

**DOI:** 10.1371/journal.pone.0242322

**Published:** 2020-12-09

**Authors:** Chenhao Qian, Yuanhe Qiu, Ziyang He, Weiwei Mu, Yongmeng Tang, Haijun Wang, Mengmeng Xie, Weixi Ji

**Affiliations:** 1 School of Mechanical Engineering, Jiangnan University, Wuxi, Jiangsu, China; 2 Jiangsu Key Laboratory of Advanced Food Manufacturing Equipment and Technology, Jiangnan University, Wuxi, Jiangsu, China; 3 China Unicom Guangzhou Branch, Guangzhou, Guangdong, China; 4 UnionPay International Headquarter, Pudong New District, Shanghai, China; University of Akron, UNITED STATES

## Abstract

In this paper, a (Fe_50_Mn_30_Co_10_Cr_10_)_100-x_C_x_ high-entropy alloy (HEA) was successfully prepared by using the vacuum arc melting method. The peak shape analysis of the X-ray diffraction patterns, the EBSD observations, and the EDS spectra of the alloys with different compositions show that the characteristics of the dendrites and the hard phase, Cr_23_C_6_, into the initial single-phase face-centered cubic (FCC) matrix becomes gradually visible as the carbon content increases from 0 to 4%. The crystal phase variations lead to a non-linear orientation of the microstructure, to a refinement of the grains, and to a higher elastic modulus. This study presents the solid saturation limit of the interstitial carbon atoms in such alloys and establishes an empirical relation between an alloy’s elastic modulus and its carbon content.

## 1. Introduction

As a kind of super-solid solution different from the traditional alloys [1], high-entropy alloys (HEAs) are composed of a variety of principal metal elements or non-metal elements, which are present approximately in an equal ratio. The characteristics of the HEAs are determined by each principal element. In recent years, due to their high strength [2], high hardness [3], high electrical resistivity [4], good wear resistance [5], corrosion resistance [6], soft magnetic properties [7], and thermal stability [8], the use of HEAs in novel applications such as mold production, cutting tools, hard coatings, biomedical materials, thermoelectric materials, and superconducting materials has fostered a large interest. In this paper, a (Fe_50_Mn_30_Co_10_Cr_10_)_100-x_C_x_ HEA was designed and prepared via the vacuum arc melting method. The effects of the interstitial carbon atoms on its microstructure and mechanical properties were studied to provide guidance for the development of such metallic composites.

## 2. Experimental materials and methods

**Fig 1:**
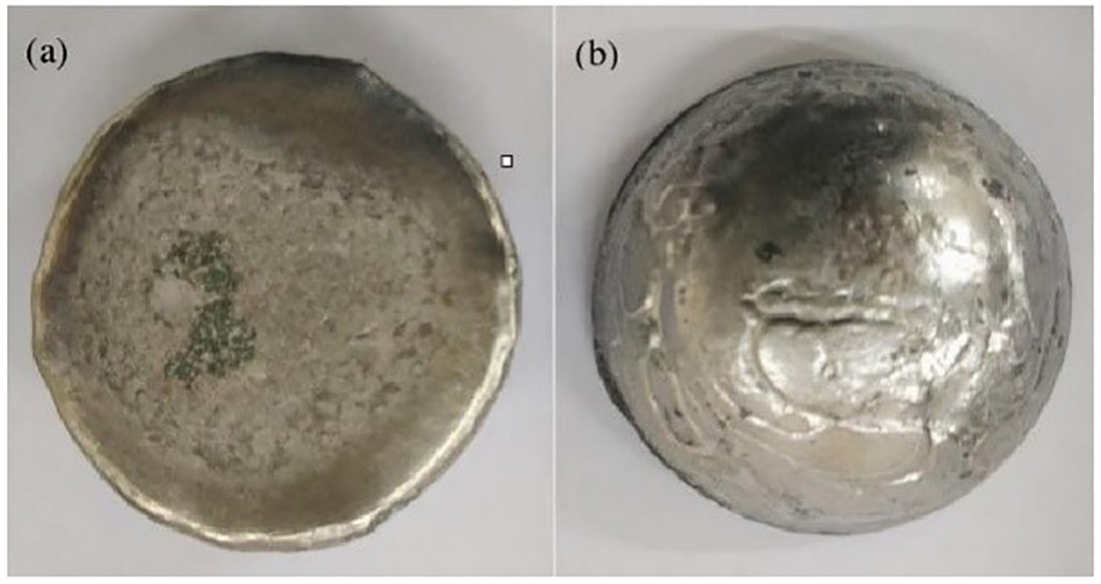
Morphology of the (a) forward and (b) reverse melting (Fe_50_Mn_30_Co_10_Cr_10_)_100-x_C_x_ alloy ingot.

Fe, Mn, Co, and Cr powders were weighed and mixed according to a mass ratio of 5:3:1:1 (Beijing Goodwill, 500 mesh, 99.9%). The graphite powder was sequentially weighed and incorporated into the mixed metal powder in different mass fractions (2%, 4%, 6%, 8%, and 10%) to obtain the starting material of the alloys (Sigma-Aldrich, 45um, 99.99%). These samples were weighed separately, placed in a powder pressing instrument, and pressed into a block under a load of 5 KPa. The block was washed with ethanol in an ultrasonic bath and dried. Then, it was placed into a copper crucible in a vacuum arc smelting furnace, which was initially evacuated and then filled with argon gas. The arc was ignited (the maximum melting current was maintained below 800 A) and the alloy was melted. Via electromagnetic stirring, the molten alloy solution was stirred uniformly and the forward and reverse melting were carried out repeatedly to obtain an ingot, as shown in Fig 1. The alloy ingot specimens were sanded, polished with a diamond paste, and the polished surface was etched with aqua regia. In this study, the observation of the grain orientation and the elemental energy spectral analysis were carried out by using a scanning electron microscope (JSM-6490LV-type SEM, JEOL™), backscattered electron diffraction (EBSD), and an energy dispersive spectrometer (EDS). An X-ray diffractometer (D/MAX2500V-type XRD, Rigaku™) was adopted to perform the phase analysis of the specimens in a scanning range of 20°–90° and with a scanning step of 0.052°. A nanoindenter (NanoIndenter G200, MTS™) was used to characterize the nanoscale mechanical properties of the specimens.

## 3. Results and analysis

**Fig 2:**
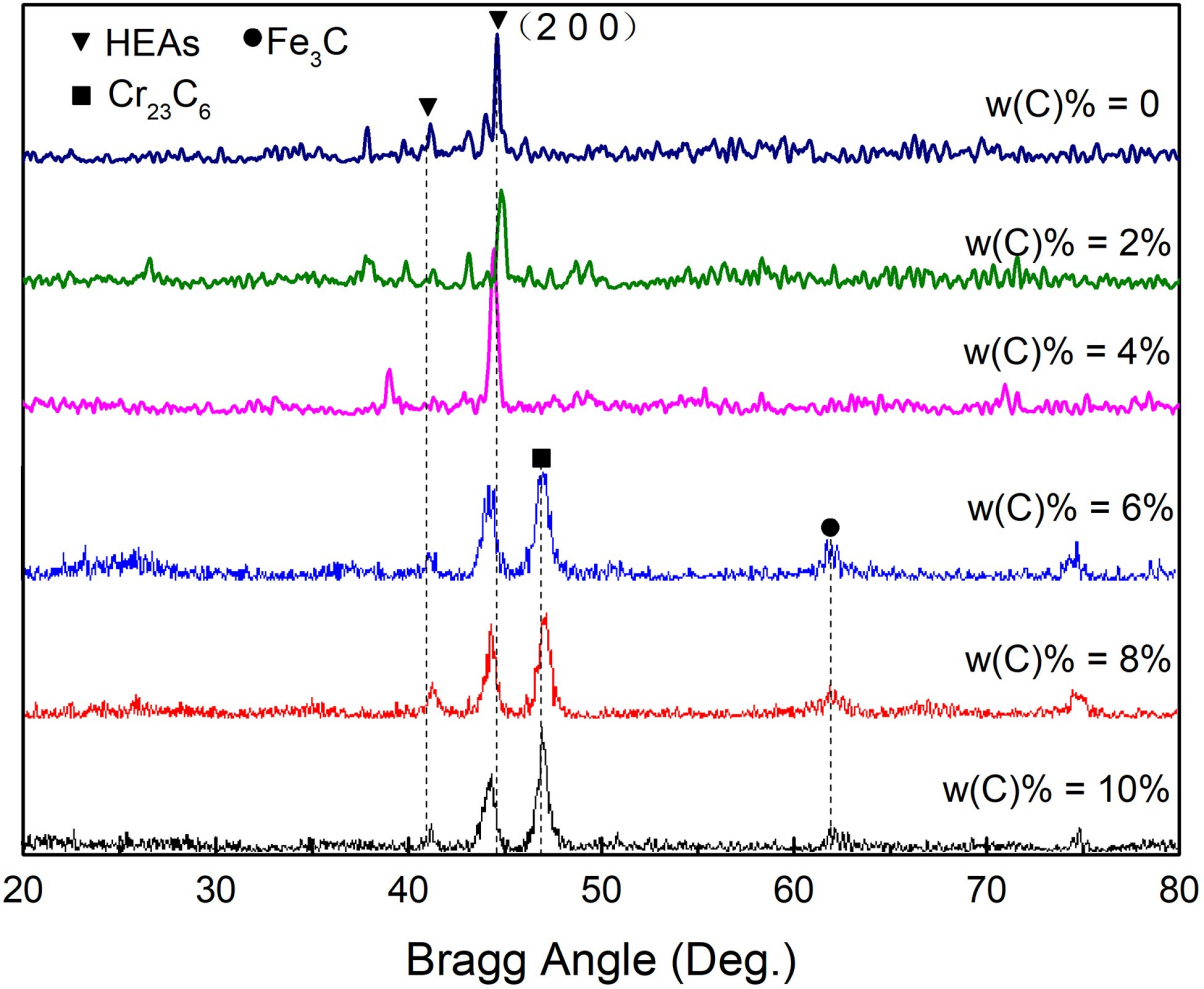
XRD diffraction pattern of the alloys with different formulations.

Fig 2 shows the XRD diffraction patterns of the carbon-free alloys and the as-cast HEAs. The XRD diffraction peak shapes of the alloys with different formulations indicate that the HEAs have a single-phase FCC crystal structure. Upon a gradual increase in the carbon content, the diffraction peak of the (200) crystal plane shifts to the left and this suggests that a large number of carbon atoms dissolves in the FCC lattice. As the carbon content, *w(C)%*, reaches 4%, the characteristic peak of the cementite appears, indicating its formation. One can speculate that the carbon atoms reach their solution limit in the voids of the FCC lattice when the solid solubility is 4%. As the carbon content continues to increase, the intensity of the main peak (200) in the FCC decreases, reflecting the precipitation of the carbides.

**Fig 3:**
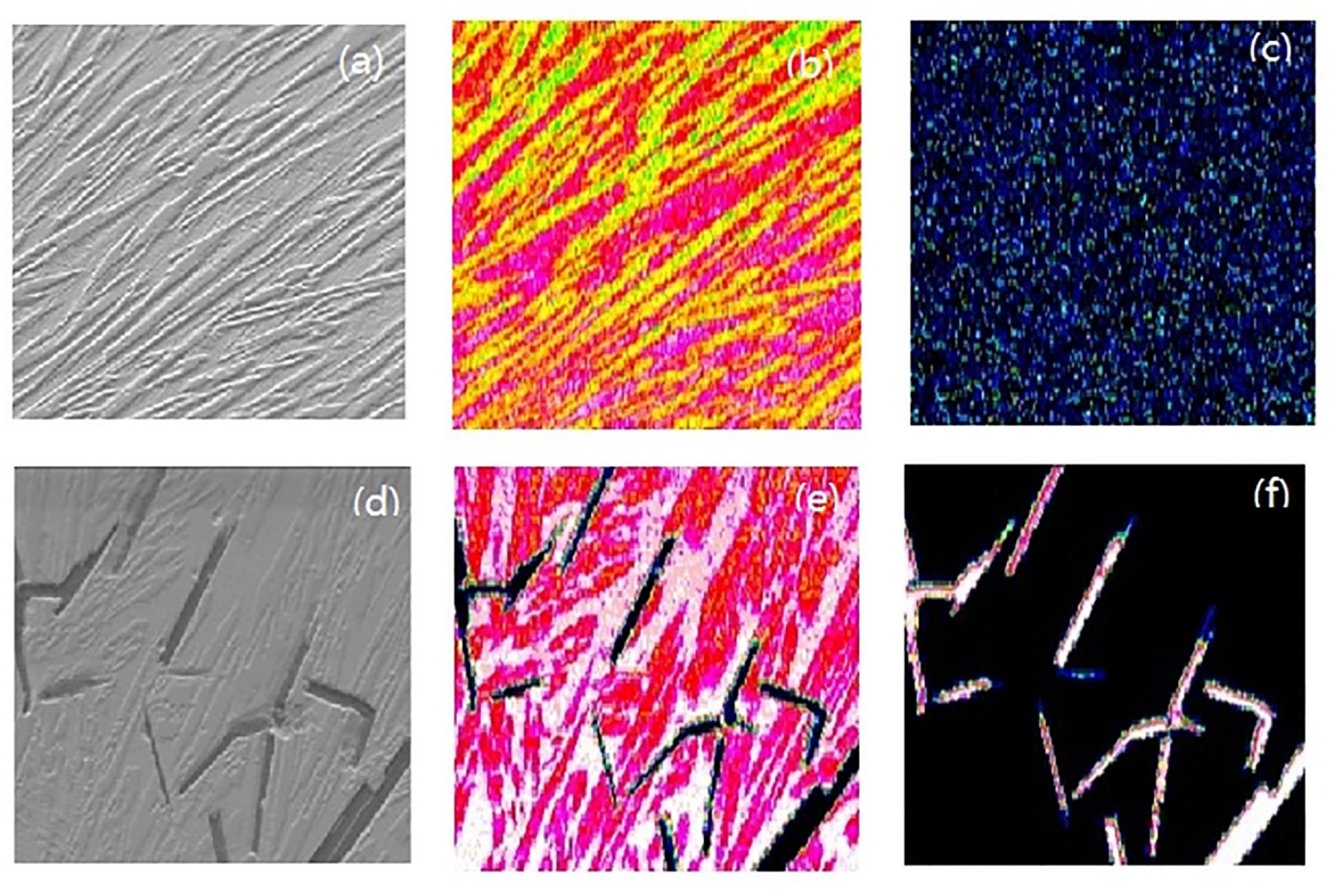
SEM image (a) and EDS element mappings of Fe and C (b-c) of the as-cast HEAs sample with 2% w(C); SEM image (d) and distribution of Fe and C (e-f) of the as-cast HEAs sample with 8% w(C).

The EBSD orientation profile and the histogram shown in Fig 3 further prove the single-phase characteristic of the as-cast HEAs. The HEAs with *w(C)%* smaller that 4% show a similar average matrix grain sizes (about 95 μm) upon performing the calculations based on the linear intercept method [9]. This indicates that the effects of the increasing interstitial carbon atoms on the matrix grain size is not significant and that no significant characteristics of the carbides can be observed in the microstructure (Fig 3a and 3b). However, the HEAs with *w(C)%* higher than 4% present obvious characteristics of dendrites and carbides (Fig 3c). An elemental analysis via EDS was performed in the regions where the carbides are present and their composition appears to be Cr_23_C_6_. According to the aforementioned results, one can speculate that the increasing content of interstitial carbon atoms initiates an increase in the dendrite quantity when the *w(C)%* is lower than 4%. When the *w(C)%* is higher than 4%, the carbon atoms in the matrix begin to saturate and the excessive carbon atoms react with the matrix to form Cr_23_C_6_, which starts to precipitate. The distribution of Cr_23_C_6_ at the grain boundary of the matrix directly leads to a decrease in the grain size of the as-cast alloy. Moreover, as shown in the histogram (Fig 3d, 3e and 3f), the orientation of the HEAs becomes random when the *w(C)%* increases from 2% to 4% and this is probably due to the low amount of dendrites in the alloy. However, the grain orientation of the entire system become observably equiaxed when the *w(C)%* reaches 8%, reflecting the effect of the carbides, which are formed at the grain boundaries.

**Fig 4:**
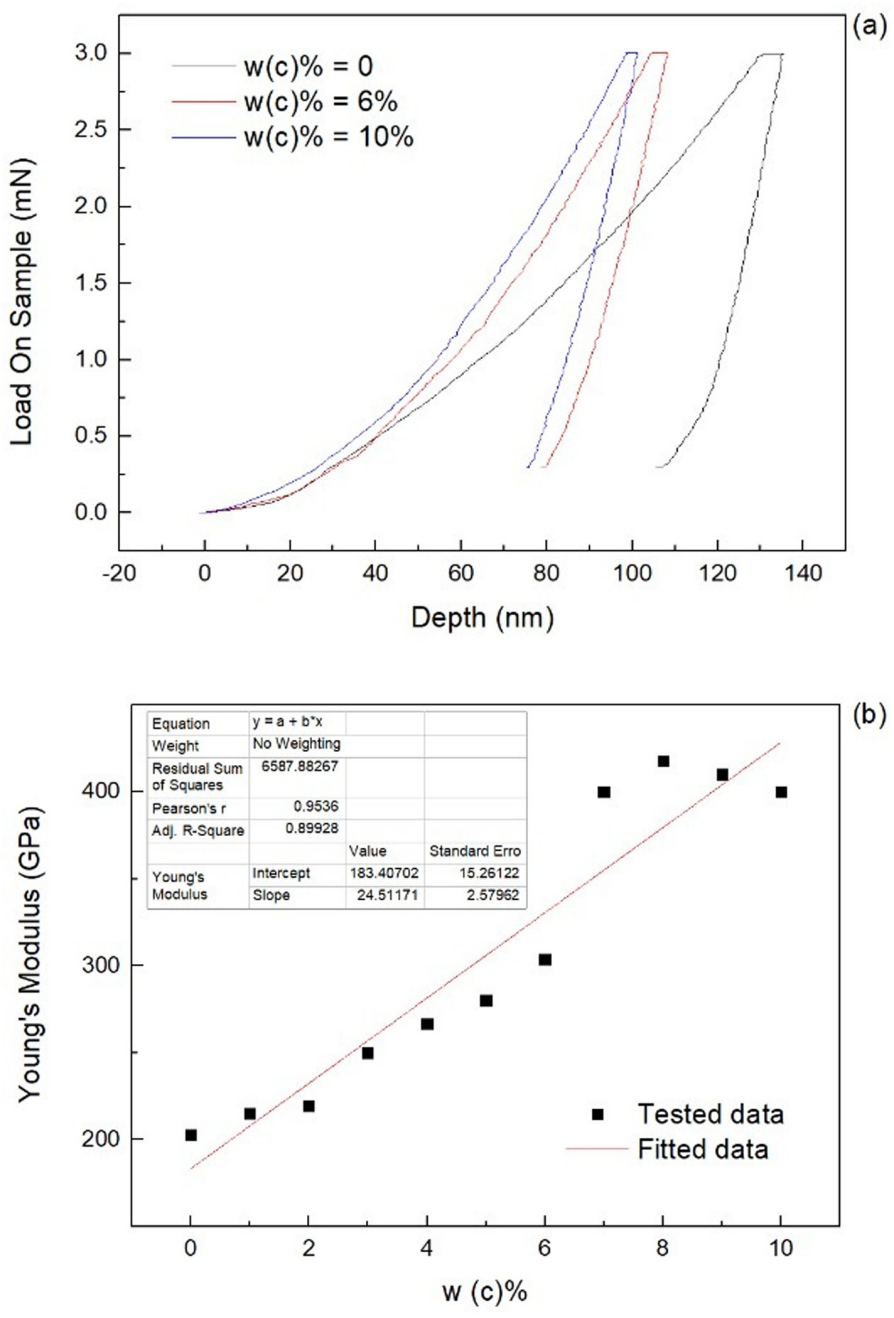
Experimental data obtained via nanoindentation: (a) load-indentation depth curve and (b) elastic modulus as a function of *w(C) %*.

The effect of the *w(C)%* on the alloy’s mechanical properties was studied by converting the load-indentation depth data measured via the nanoindentation technique (Fig 4a) into an equivalent elastic modulus (Fig 4b). These results show that there is an approximate linear relationship between the carbon content, *w(C)%*, and the elastic modulus, E, which can be defined by the following mathematical expression: *E* = 183.4+24.5×*w(C)%*×100. This linear positive correlation between *E* and *w(C)%* is attributed to the combination of the dispersion-strengthening effect of the generated Cr_23_C_6_ when compared to the matrix. For the Young’s Modulus stagnation at approximate 400 GPa, it is supposed to attributed to following reasons: 1) carbon saturation rate is around 6%wt level, so after this threshold, the carbides generated and located at the grain boundaries. This caused a huge jump for elastic modulus comparing to previous samples. 2) after large amount of carbides generation, the carbide is not the main variable to affect the overall mechanical strength, so then stagnation from 6%wt to 10%wt samples at 400GPa was observed. Moreover, the strengthening, due to the grain size reduction of Cr_23_C_6_, inhibits the grain growth of the as-cast HEAs matrix. Although the hard phase (Cr_23_C_6_) with a random FCC structure [10] shows enhancements on the strength, hardness, and stiffness performances of the HEAs, it tends to weaken the alloy ductility.

## 4. Conclusions

1. The solid solubility of the interstitial carbon atoms in the matrix lattice of a FeMnCoCr alloy measures 4%.
2. The increase in the quantity of Cr_23_C_6_ formed by the presence of C and Cr in the HEAs can modify the grain orientation of the alloy system.
3. The elastic modulus, *E*, and the carbon content, *w(C)%*, exhibits an approximately linear positive correlation.

## References

[1] Yeh J.W., et al, Nanostructured high-entropy alloys with multiple principal elements: Novel alloy design concepts and outcomes. ADVANCED ENGINEERING MATERIALS, 2004 6(5): p. 299–303.

[2] Mu S., et al, Electronic transport and phonon properties of maximally disordered alloys: From binaries to high-entropy alloys. JOURNAL OF MATERIALS RESEARCH, 2018 33(19): p. 2857–2880.

[3] Sarker P., et al, High-entropy high-hardness metal carbides discovered by entropy descriptors. NATURE COMMUNICATIONS, 2018 9(4980). 10.1038/s41467-018-07160-7 30478375 PMC6255778

[4] Lu Z., et al, Deformation Behavior and Toughening of High-Entropy Alloys. ACTA METALLURGICA SINICA, 2018 54(11SI): p. 1553–1566.

[5] Li Y., et al, Design of Fe-Based Bulk Metallic Glasses with Improved Wear Resistance. ACS APPLIED MATERIALS & INTERFACES, 2018 10(49): p. 43144–43155. 10.1021/acsami.8b11561 30422626

[6] Huang K., et al, Wear and Corrosion Resistance of Al0.5CoCrCuFeNi High-Entropy Alloy Coating Deposited on AZ91D Magnesium Alloy by Laser Cladding. ENTROPY, 2018 20(91512). 10.3390/e20120915 PMC7512501 33266639

[7] Jiao D.L., et al, Thermal stability, magnetic and magnetocaloric properties of Gd55Co35M10 (M = Si, Zr and Nb) melt-spun ribbons. CURRENT APPLIED PHYSICS, 2018 18(12): p. 1523–1527.

[8] Xing Q., et al, High-Throughput Screening Solar-Thermal Conversion Films in a Pseudobinary (Cr, Fe, V)-(Ta, W) System. ACS COMBINATORIAL SCIENCE, 2018 20(11): p. 602–610. 10.1021/acscombsci.8b00055 30350567

[9] Jerban S. and Elkoun S., Novel linear intercept method for characterizing micropores and grains in calcium phosphate bone substitutes. MATERIALS CHARACTERIZATION, 2016 119: p. 216–224.

[10] Hirota K., et al, Simultaneous synthesis and consolidation of chromium carbides (Cr3C2, Cr7C3 and Cr23C6) by pulsed electric-current pressure sintering. MATERIALS SCIENCE AND ENGINEERING A-STRUCTURAL MATERIALS PROPERTIES MICROSTRUCTURE AND PROCESSING, 2005 399(1–2): p. 154–160.

